# NASA Sponsors Symposium on Remote Sensing and Control of Insect-Transmitted Diseases

**DOI:** 10.3201/eid0201.960118

**Published:** 1996

**Authors:** Michael Braukus, Raj Khanna

**Affiliations:** * National Aeronautics and Space Administration, Washington, D.C., USA † Third World Foundation of North America, College Park, Maryland, USA


epidemiologic surveillance once a strain is identified, and the rapid exchange of information globally. The following immunologic and molecular questions were addressed as well: What basic research advances would allow us to respond more rapidly after the next human pandemic strain is detected? Is the presence of novel influenza A virus in pigs a predictor of the next influenza pandemic? Is an H2 influenza virus the next human pandemic subtype or are H7 viruses equally possible? Also discussed were the practical issues of vaccine needs, production, and distribution.

Conference participants then reviewed international pandemic plans and the U.S. pandemic plan being prepared by the Federal Interagency Group on Pandemic Preparedness.

**Dominick A. Iacuzio**
Division of Microbiology and Infectious Diseases
National Institutes of Health
Bethesda, Maryland, USA

### Course Offered on Clinical and Pathologic Features of Emerging Infections

The Armed Forces Institute of Pathology (AFIP), Emory University, and the Centers for Disease Control and Prevention (CDC) are cosponsoring a course on emerging and reemerging pathogens. The course will be taught in Atlanta, Georgia from April 27 to May 1 and will discuss the epidemiology, clinical features, pathology, and pathogenesis of such diseases as plague, Lyme disease, Kaposi sarcoma, microsporidiosis, Buruli ulcer, ehrlichiosis, hantavirus pulmonary syndrome, and Ebola virus infection. Emerging drug resistance in pneumococci and other streptococcal infections will also be discussed.

The course is designed for pathologists, epidemiologists, infectious disease physicians, veterinarians, microbiologists, parasitologists, and others interested in the pathology as well as the emergence of infectious diseases. The course, to be held at the Emory Conference Center Hotel, will provide 38 hours of Category I CME credit and will consist of 32 hours of lectures with open discussion periods, 6 hours of glass and color slide review, and a visit to CDC laboratories. For more information, contact the course director, Center for Advanced Medical Education, AFIP, Washington, D.C. 20306-6000 (phone: 800-577-3749 or 301-295-7921; fax: 301-427-5001).

**C. Robert Horsburgh, Jr.**
Emory School of Medicine
Atlanta, Georgia, USA

### NASA Sponsors Symposium on Remote Sensing and Control of Insect-Transmitted Diseases

Health officials and disease control experts met November 28-30 in Baltimore, Maryland, for a symposium on the use of satellites to monitor and control insect-transmitted diseases.

Sponsored by the National Aeronautics and Space Administration (NASA) and the Third World Foundation of North America, the symposium was held to inform government officials from various countries of NASA's scientific and technologic capabilities for detecting, monitoring, and improving the control of diseases. Health ministers and medical directors from more than 20 countries, including Bangladesh, Belize, China, Ghana, Indonesia, Kenya, Malaysia, Nigeria, Peru, and Rwanda, attended.

The symposium featured discussions on the economics of disease surveillance, deforestation, and urbanization. The keynote address, "The resurgence of vector-borne infectious diseases as major public health problems in the 1990s," was given by Duane Gubler, director, Division of Vector-Borne Infectious Diseases, National Center for Infectious Diseases, Centers for Disease Control and Prevention (CDC). Participants also discussed possible joint activities between NASA and interested countries. Further information can be obtained from NASA's Office of Life and Microgravity Sciences, Washington, D.C., which manages the agency's global monitoring and human health research program in conjunction with the National Institute of Allergy and Infectious Diseases, National Institutes of Health, and CDC.

**Michael Braukus**
National Aeronautics and Space Administration
Washington, D.C., USA

**Raj Khanna**
Third World Foundation of North America
College Park, Maryland, USA

Health officials and disease control experts met November 28-30 in Baltimore, Maryland, for a symposium on the use of satellites to monitor and control insect-transmitted diseases.

Sponsored by the National Aeronautics and Space Administration (NASA) and the Third World Foundation of North America, the symposium was held to inform government officials from various countries of NASA's scientific and technologic capabilities for detecting, monitoring, and improving the control of diseases. Health ministers and medical directors from more than 20 countries, including Bangladesh, Belize, China, Ghana, Indonesia, Kenya, Malaysia, Nigeria, Peru, and Rwanda, attended.

The symposium featured discussions on the economics of disease surveillance, deforestation, and urbanization. The keynote address, "The resurgence of vector-borne infectious diseases as major public health problems in the 1990s," was given by Duane Gubler, director, Division of Vector-Borne Infectious Diseases, National Center for Infectious Diseases, Centers for Disease Control and Prevention (CDC). Participants also discussed possible joint activities between NASA and interested countries. Further information can be obtained from NASA's Office of Life and Microgravity Sciences, Washington, D.C., which manages the agency's global monitoring and human health research program in conjunction with the National Institute of Allergy and Infectious Diseases, National Institutes of Health, and CDC.

